# The Glycaemic and Insulinaemic Response of Pasta in Chinese and Indians Compared to Asian Carbohydrate Staples: Taking Spaghetti Back to Asia

**DOI:** 10.3390/nu13020451

**Published:** 2021-01-29

**Authors:** Stefan Gerardus Camps, Joseph Lim, Melvin Xu Nian Koh, Christiani Jeyakumar Henry

**Affiliations:** 1 Clinical Nutrition Research Centre (CNRC), Singapore Institute of Food and Biotechnology Innovation (SIFBI), Agency for Science, Technology and Research (A*STAR), Centre for Translational Medicine, Yong Loo Lin School of Medicine, National University of Singapore, 14 Medical Drive #07-02, MD 6 Building, Singapore 117599, Singapore; joseph_lim@sifbi.a-star.edu.sg (J.L.); melvin_koh@sifbi.a-star.edu.sg (M.X.N.K.); 2 Department of Biochemistry, Yong Loo Lin School of Medicine, National University of Singapore, S14 Level 5, Science Drive 2, Singapore 117543, Singapore

**Keywords:** Asia, glycaemic index, glucose, insulin, pasta, spaghetti, food structure, rice, noodles

## Abstract

In this study, we compared the metabolic properties of the Asian staples rice and noodles, which are typically high in glycaemic index (GI), to two types of spaghetti. It is hypothesised that pasta can be a healthy replacement, particularly amongst the Asian population. Thirty Chinese and Indian subjects (17 men, 13 women; BMI: 18.5–25 kg/m^2^) participated in this randomised crossover trial. On seven occasions, they consumed a glucose reference drink (3 times), white rice, wheat-based mee pok noodles, semolina spaghetti and wholegrain spaghetti. Blood samples were taken to measure glucose and insulin response over a period of 3 h. The current evaluation showed that semolina spaghetti and wholegrain spaghetti can be classified as low GI products, with a GI of 53 and 54, respectively, significantly lower than wheat based mee pok noodles (74) and rice (80) (*p* < 0.005). In addition, both spaghettis had a lower insulin response compared to rice (*p* < 0.05). Furthermore, there was no difference in glucose or insulin response between semolina and wholegrain spaghetti. After controlling for gender, ethnicity, fat and fat free mass (kg), the glucose and insulin results did not change. In conclusion, wheat-based pasta can be helpful to modify the carbohydrate-rich Asian diet. Notably, there was no effect of gender, ethnicity and body composition on the glycaemic and insulinaemic response. We speculate that the starch-protein structure as a result of the spaghetti production process is a major driver of its favourable metabolic properties.

## 1. Introduction

Diabetes is a rapidly growing health problem globally and even at a healthy BMI, Asians are more susceptible to the onset of prediabetes and show a more rapid decline to diabetes [1,2,3]. In addition, weight gain plays an important roles in the global diabetes epidemic, and the fast increase in the prevalence of obesity is reported in many Asian countries too [4]. Lifestyle changes, including improved diets and physical activity, may be crucial to prevent, delay or reverse obesity and health related diseases like diabetes [5]. The glycaemic index (GI) is a classification method of carbohydrate foods based on the effect on postprandial blood glucose levels. [6] The consumption of low GI foods lowers the risk for the onset of pre-diabetes and impaired glucose metabolism as it will moderate post-prandial glucose levels, glucose fluctuation, and subsequently insulin levels [7,8,9,10]. Furthermore, consumption of low glycaemic response diets are linked to favourable changes in cardiovascular disease markers [11]. 

Out of the more than 50,000 edible plants, maize, rice and wheat provide over half of the global energy intake. White rice is classified as high GI and makes up a large part of the daily carbohydrate consumption in South and East Asia [12]. A recent systematic review and meta-analysis by Hu et al. showed a clear relationship between the amount of white rice consumed and the risk of diabetes [13]. In addition, higher levels of rice intake were more strongly associated with the diabetes risk in Asian compared to Western populations [13]. 

Wheat is the third most important crop in terms of global production [14], and it has the distinctive trait that it can be cultivated within a large latitudinal range, including elevated regions and tropical climates [15]. Industrialisation and westernisation have led to an increase in consumption and production demand is on the rise due to the potential to produce unique food products. In particular, the gluten protein fraction gives wheat its special properties to be used for foods like bread, pastries, noodles and pasta [16]. These wheat derived food products come in a whole range of GI; e.g. white bread is generally classified as high GI, wheat roti as medium GI and many types of pasta can be classified as low GI [17]. The production process of pasta gives rise to its distinct starch structure and its rheological characteristics [18], which may play a significant role in determining the rate of starch digestion [19] and influencing the glycaemic response to its ingestion [20].

The preferred and most often used raw material for good quality pasta is durum wheat semolina (Triticum turgidum ssp. durum). During the milling of wheat, the endosperm is separated from the bran and germ and subsequently ground into flour that can be used as an ingredient for many food products. The cracked endosperm particles of durum wheat are called semolina. The removal of the bran and germ result in a reduction of micronutrients and fibre content compared to wholegrain products [21,22,23]. 

In this study, the glycaemic and insulinaemic response was compared between wheat-based pasta (spaghetti), wheat based mee pok and rice and, additionally, whether there will be an effect of gender, ethnicity (Chinese and Indian) and body composition on the metabolic response. It is hypothesised that pasta can be a healthy replacement for rice and noodles, particularly amongst the Asian population. Additionally, wholegrain pasta and semolina pasta were compared to assess whether the type of flour used or the pasta production process is more important for the potential beneficial effect. It is hypothesised that pasta will have a favourable metabolic response compared to the Asian staples. Moreover, it is hypothesised that the unique food structure of pasta as a result of the production process is the main driver of this effect.

## 2. Materials and Methods

### 2.1. Subjects

Thirty-two healthy Chinese and Indian subjects (17 men, 15 women), between 21–40 years, were recruited by a variety of methods, which included online advertisement and flyers and posters around the university campus. Subjects underwent an initial screening and measurements included anthropometry (height, weight, waist and hip circumference), fat percentage via bio impedance analysis (Tanita), blood pressure, resting heart rate and fasting blood glucose via finger prick (HemoCue® 201+ Glucose analyser (HemoCue Ltd, Dronfield, UK)). Additionally, a questionnaire on general health was completed. Subjects were non-smokers, had a healthy BMI, had a normal blood pressure, were not glucose-6-phospahate dehydrogenase deficient, had no medical conditions, nor were taking medications known to affect glycaemia (glucocorticoids, thyroid hormones, thiazide diuretics) and were not allergic to the test foods. Two subjects were unable to commit to all test sessions and withdrew from the study; as a result, thirty subjects completed all treatments and were included for analysis. 

The study was conducted at the Clinical Nutrition Research Centre (CNRC), Singapore. All subjects gave their informed consent for inclusion before they participated in the study. The study was conducted in accordance with the Declaration of Helsinki, and the protocol was approved by the National Healthcare Group Domain Specific Review Board (NHG DSRB Reference Number: 2018/00622). This trial was registered at Clinicaltrials.gov (NCT03646812).

### 2.2. Study Design

The study consisted of seven dietary test treatments in a randomised, cross-over design: three glucose reference drinks, jasmine white rice (Royal Umbrella), teochew mee pok noodles (Fortune), semolina spaghetti (Barilla) and wholegrain (wholewheat) spaghetti (Barilla). Subjects attended seven test sessions of around 3.5 h, separated by a wash-out period of at least one day. Each test session consisted of a test meal that had to be consumed within 12 min and 3 h measurement of metabolic blood parameters. Subjects arrived at the test centre around 8:30 following an overnight fast of at least 10 h. In addition, subjects were instructed to refrain from strenuous physical activity and alcohol on the day before the test and were only allowed to eat the provided foods throughout the test sessions. Online computer software (Social Psychology Network, Middletown, CT, USA) was used for simple randomisation of the sequence of the treatment diets (http://www.randomizer.org/) [24].

### 2.3. Treatment Meals

During each of the seven test sessions, subjects consumed one of the following test treatments: jasmine white rice (Royal Umbrella), teochew mee pok noodles (Fortune), semolina spaghetti (Barilla), wholegrain (wholewheat) spaghetti (Barilla) or glucose reference drink (50 g anhydrous glucose dissolved in 250 mL water). All the test foods and the reference drinks were given in portions containing 50 g of available carbohydrates. After subjects finished the meal, they were asked to answer the following question using a 100 mm visual analogue scale (VAS) ranging from dislike extremely (0 mm) to like extremely (100 mm), with neither like nor dislike in the middle (50 mm): “Please rate your overall liking of the test meal.” The total meal characteristics and macronutrient composition of the test foods is shown in Table 1 and the preparation for each test meal is shown in Table 2. 

### 2.4. Metabolic Plasma Parameters

During each test session, 5 μL blood samples were collected via finger pricks for 3 h and blood glucose was analysed immediately using the HemoCue® 201+ Glucose analyser; two capillary blood samples were collected five minutes apart to measure baseline blood glucose concentrations (0 min), and following the test meal, blood samples (5 μL) were collected at 15, 30, 45, 60, 90, 120, 150 and 180 min. 

In addition, 500 μL capillary blood was collected in an ethylenediaminetetraacetic acid (EDTA) coated container at 0, 30, 60, 90, 120, 150 and 180 min for insulin analysis. The blood was centrifuged using a micro-centrifuge at 10,000 G for 10 min at 4 °C, and the separated plasma samples were transferred into Eppendorf tubes and stored at −80 °C until analysis. Insulin concentration in the plasma samples (µU/mL) was analysed using the Cobas e411 electrochemiluminescence immunoassays analyser (Roche, Hitachi, Indianapolis, IN, USA). A cross-over design with a minimum of eight subjects would be sufficient to detect a 15% change in area under the glucose curve with a power of 0.85 at a significance level of 0.05 [25].

### 2.5. Statistics

All statistical analyses were performed using Statistical Package for the Social Sciences (SPSS) version 23 (IBM Corp. Armonk, NY, USA). Data and figures were processed in a Microsoft Excel spreadsheet (Microsoft Corp, Redmond, WA, USA). Values were presented as mean ± SEM unless otherwise stated. Prior to statistical analysis, the normality of the data was assured using the Shapiro–Wilks test. 

The primary outcome of this study was the glycaemic and insulinaemic response, compared between wheat-based pasta (spaghetti), wheat based mee pok and rice. Fluctuations of glucose and insulin were calculated according to a fasted baseline sample and the post-prandial response was expressed as the incremental area under the curve (iAUC) calculated using the trapezoidal rule [26,27]. Using the iAUC values, the GI and insulinaemic index (II) were calculated using the following formula (1) (FAO/WHO, 1998) in accordance with the International Organization for Standardization (ISO) 26642:2010 guidelines [28,29].

(1) $$GI\;or\;II=\;\frac{iAUC\;for\;the\;test\;food}{average\;iAUC\;for\;glucose\;reference}\;\times \;100$$

Repeated measures analysis of variance (ANOVA) with post hoc analysis correcting for family wise error using Bonferroni correction was used to determine differences in glycaemic and insulinaemic response. Additionally, a linear mixed model with type of food as fixed factor and random subject effect was performed to investigate the overall effect of food type on GI and II. Additional variables such as gender, ethnicity, fat mass (FM) and fat free mass (FFM) were included into the model to study the effect of food type on GI or II while controlling for these possible confounding variables individually. Alpha (α) was set at 0.05 for statistical analyses. 

Twenty-seven complete datasets were used for the final analysis (datasets of 3 subjects were removed because of statistical outlier based on SPSS interquartile rules). Of the 27 subjects, 15 were Chinese (10 men, 5 women) and 12 were Indian (5 men, 7 women).

## 3. Results

The subject characteristics can be found in Table 3.

**Figure 1:**
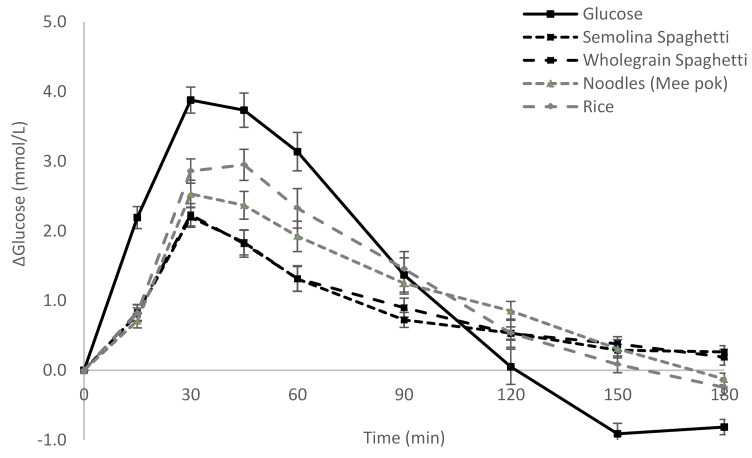
Postprandial change in glucose (mean ± standard error).

**Figure 2:**
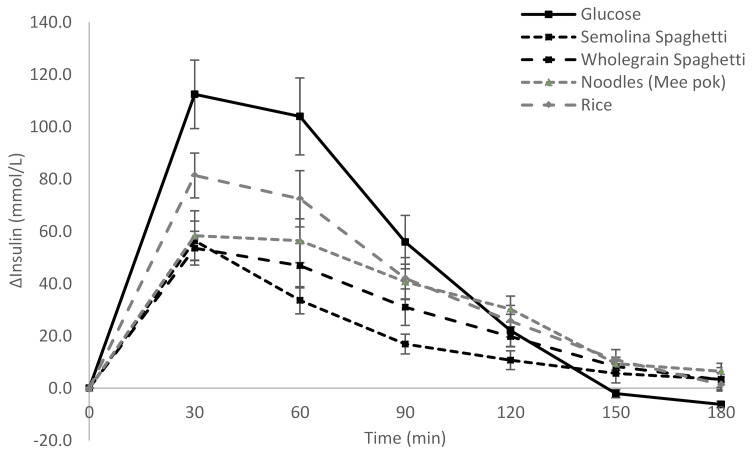
Postprandial change in insulin (mean ± standard error).

The change in postprandial change in glucose and insulin over time (180 min) can be seen in Figure 1 and Figure 2. It is clear that both semolina and wholegrain spaghetti have a lower glucose and insulin response over time as compared to rice and noodles. In particular, the glucose response is lower at 45, 60 and 90 min after the start of the meal for both the spaghettis as compared to noodles and rice (*p* < 0.05) (Figure 1). The insulin response for the spaghettis is lower than rice at 30 min and 60 min (*p <* 0.05). Furthermore, the semolina spaghetti has a lower insulin response than wholegrain spaghetti at 90 min (17 ± 4 vs. 30 ± 6, *p* < 0.05) and 120 min (10 ± 3 vs. 20 ± 3, *p* < 0.05) (Figure 2).

**Figure 3:**
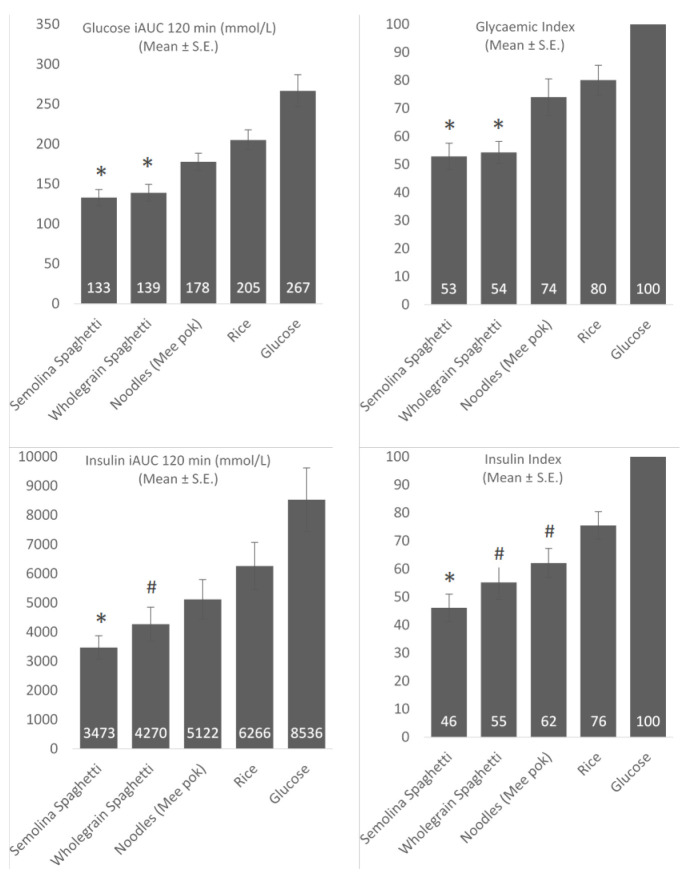
Glycaemic and insulinaemic response as incremental area under the curve (iAUC) (mmol/L) and indexed versus the glucose treatment. Mean ± standard error * (*p* < 0.05) different from mee pok and rice, ^#^ (*p* < 0.05) different from rice.

The glucose iAUC and glycaemic index (GI) can be seen in Figure 3. The glucose iAUC (120 min) was significantly lower for both semolina and wholegrain spaghetti as compared to noodles (*p* < 0.005) and rice (*p <* 0.005). Similarly, the GI of both semolina and wholegrain spaghetti was lower than the GI of noodles (*p <* 0.01) and rice (*p* < 0.001). 

The insulin iAUC and insulinaemic index (II) can be seen in Figure 3. The II of semolina spaghetti was lower than the II of noodles (*p <* 0.05) and rice (*p <* 0.001) and the II of wholegrain spaghetti was lower than the II of rice (*p <* 0.005). Table 4 shows an overview of the GI and II as mean ± standard deviation and as a median value. After controlling for gender, ethnicity, FM (kg) and FFM (kg), the glucose and insulin results did not change, indicating that ethnicity, gender and body composition did not have an effect on the observed differences between the foods. There was a significant effect of FM on the GI of the foods (*p =* 0.027), though this did not affect the differences between the foods.

There was no significant difference in overall liking between the four meals as measured based on the VAS results (Table 4). 

## 4. Discussion

The current study measured the metabolic response of pasta, rice and noodles in Chinese and Indian men and women. The results showed that the glycaemic and insulinaemic response of spaghetti pastas were lower than the response of rice and noodles. Furthermore, after controlling for the possible role of ethnicity, gender and body composition, the differences between the spaghetti pastas and rice and noodles remained the same.

The current evaluation showed that semolina spaghetti and wholegrain spaghetti can be classified as low GI products with a GI of 53 and 54, respectively. Both had a significantly lower incremental glucose response and GI as compared to wheat based mee pok noodles (74) and rice (80). In addition, the insulin response after pasta consumption compared favourably to noodles and rice. This confirmed the first hypothesis that wheat-based pasta can be helpful to modify the Asian diet. Notably, there was no significant difference in glycaemic response between the semolina wholegrain spaghetti, even with the latter containing more than double the amount of fibre. Moreover, there was a significantly lower insulin response at 90 min and 120 min and a trend for a lower overall insulin response (iAUC and II) with semolina spaghetti when compared to wholegrain spaghetti. 

The industrial production process, especially the drying temperature, extrusion and the cooking process, affect starch gelatinisation and gluten coagulation leading to the distinctive food matrix of pasta and give pasta its rheological characteristics [18] that will influence the starch digestibility and protein hydrolysis [30,31]. The covalent bonds between the proteins will influence the action of the proteolytic enzymes [30,31,32] and the compact protein structure entraps the starch granules leading to reduced or delayed access for digestive enzymes [33]. Therefore, it is speculated that the structure of pasta might have a significant role in the rate of digestion and glycaemic response [19,20].

In the current study, the amount of available carbohydrates was matched between the conditions, though the wheat-based products contained more fibre and protein than rice, and the wholegrain spaghetti contained more fibre than semolina spaghetti (> 2 ×) and mee pok noodles (> 4 ×). The stimulating effect of dietary protein on insulin has been shown repeatedly [34,35,36] and acute studies indicated that protein consumption can attenuate the glycaemic response due to the insulinotropic effect [37,38]. In the current study, no insulin stimulating effect was observed after consumption of the wheat-based products as compared to rice. The meals in this study consisted of a high carbohydrate food only as compared to other studies that would provide a protein source or amino acids in addition to the carbohydrate source. 

In general, the consumption of wholegrain products is linked to improved insulin sensitivity and a lower glycaemic response, which is partly attributed to fibre [39]. Short-term interventions studies [40] and epidemiological and prospective studies have consistently shown an association between wholegrain consumption and a reduced mortality and metabolic disease risk [41,42]. However, some studies evaluated the role of dietary fibre in lowering the GI of products, but obtained discordant results, as was observed in the current study [43,44]. Indeed, only certain types of fibre, mainly soluble (i.e., vegetable gums, derived from fruits, legumes and psyllium), may influence the GI of foods through a reduced rate of gastric emptying as they make the chyme (partly digested food coming from the stomach) more viscous [43,44]. Moreover, significant effects are obtained only in those products that naturally have a high GI (i.e., bread) [43,44,45,46]. Wheat bran is rich in insoluble fibre and the presence of bran has shown to be detrimental to pasta structure [47]. The higher content of bran and germ particles within the wholegrain pasta matrix may physically interfere with the gluten matrix and result in a more porous structure, leading to more accessible starch granules for digestive enzymes [48]. This is in line with the results of the current study as the semolina spaghetti showed an improved insulin response compared to wholegrain spaghetti. It confirms that a higher fibre content in spaghetti may not be beneficial for the metabolic response. On the other hand, this leads to the speculation that the protein-starch matrix itself, owing to the production process, is the main basis for the reduced glycaemic response of pasta and this warrants further research. This would be in agreement with the second hypothesis, which is that the food structure of the spaghetti is the main driver of its favourable metabolic properties. 

The introduction of pasta in Asia may sound counterintuitive; however, it is important to recognize that pasta and noodles are very similar foods. Interestingly, in recent years, there has been considerable interchange in the Asian region where pasta and noodles are being exchanged to formulate fusion foods and dishes. Some of these iconic fusion dishes are: Hong Kong macaroni soup, Filipino spaghetti, spaghetti Goreng (Malaysia and Indonesia), Wafu pasta (Japan) and Tom Yum spaghetti (Thailand). Furthermore, pasta consumption in Asia is projected to grow by 35% between 2015 and 2025 [49]. The VAS scores from the current study support that there is no concern for the lack of liking of pasta as there was no difference in “liking” between spaghetti, noodles and rice.

## 5. Conclusions

The current study directly compared the effect on glycaemic and insulin response of the Asian staples white rice and noodles versus wheat spaghetti in Chinese and Indian men and women. In conclusion, wheat-based pasta can be helpful to modify the carbohydrate-rich Asian diet. Notably, there was no effect of gender, ethnicity and body composition on the glycaemic and insulinaemic response differences that were measured between the foods. We speculate that the production and development process of pasta may in itself be conducive to develop low GI products. The reduced metabolic response induced by spaghetti is likely explained by the compact starch and protein network. This suggests that it may not be necessary to either include wholegrain or dietary fibre to reduce the glycaemic response of pasta-like products. The results showed that among Chinese and Indian men and women, the pasta meals would result in a lower immediate glucose response. These data can be valuable for food recommendations in the Asian population, and wheat-based pasta can be a realistic healthy alternative to replace rice and noodles in the Asian diet.

## Figures and Tables

**Table 1:** Macronutrient composition per 100 g.

	Carbohydrate	Fibre	Fat	Protein
Semolina Spaghetti	71.2	3.0	2.0	12.5
Wholegrain Spaghetti	65.7	6.5	2.5	13.0
Mee Pok	54.1	1.4	1.1	10.1
White rice	79.7	0.5	0.7	6.7

**Table 2:** Test meal preparation details.

Semolina Spaghetti	71 g of spaghetti 1 L of water 7 g of salt	Boil 1 L of water with 7 g of salt. When boiling add 1 portion of spaghetti and boil for 8 min. Drain and consumed immediately.
Wholegrain Spaghetti	76 g of spaghetti 1 L of water 7 g of salt	Boil 1 L of water with 7 g of salt. When boiling add 1 portion of spaghetti and boil for 8 min. Drain and consumed immediately.
Mee pok noodles	92.4 g of noodles 1 L of water	Boil 1 L of water. When boiling, add 1 portion of noodles and cook for 45 s. Drain and consumed immediately.
White Rice	63 g of rice 150 mL of water	Cook rice with water in rice cooker.

**Table 3:** Subject characteristics (*n* = 27).

	(Mean ± S.D.)
Age (years)	26.4 ± 5.4
Height (cm)	172.6 ± 9.9
Weight (kg)	68.0 ± 12.0
BMI (kg/m)	22.6 ± 2.1
Body Fat %	24.7 ± 7.2

S.D.: standard deviation, BMI: body mass index.

**Table 4:** Glycaemic Index (GI), Insulinaemic Index (II) and overall liking of the four treatment meals (mean ± standard deviation).

	GI (Mean ± S.D.)	GI (Median)	II (Mean ± S.D.)	II (Median)	Liking
Semolina Spaghetti	52.9 ± 24.0	53.1	46.0 ± 25.4	42.5	54.3 ± 23.9
Wholegrain Spaghetti	54.3 ± 21.9	51.4	55.1 ± 31.5	45.7	51.5 ± 26.1
Noodles (Mee pok)	74.0 ± 31.3	64.7	61.5 ± 24.0	57.8	46.2 ± 27.8
Rice	80.1 ± 25.8	78.9	75.0 ± 23.8	71.4	45.9 ± 25.3

## Data Availability

The data presented in this study can be requested from the corresponding author.
